# Tuberculosis infection in rural labor migrants in Shenzhen, China: Emerging challenge to tuberculosis control during urbanization

**DOI:** 10.1038/s41598-017-04788-1

**Published:** 2017-06-30

**Authors:** Xiangwei Li, Qianting Yang, Boxuan Feng, Henan Xin, MingXia Zhang, Qunyi Deng, Guofang Deng, Wanshui Shan, Jianrong Yue, Haoran Zhang, Mufei Li, Hengjing Li, Qi Jin, Xinchun Chen, Lei Gao

**Affiliations:** 1 MOH Key Laboratory of Systems Biology of Pathogens, Institute of Pathogen Biology, and Center for Tuberculosis, Chinese Academy of Medical Sciences and Peking Union Medical College, Beijing, 100730 China; 2 Guangdong Key Laboratory for Emerging Infectious Diseases, Shenzhen Key Laboratory of Infection & Immunity, Shenzhen Third People’s Hospital, Shenzhen, China; 3 Department of Pathogen Biology, Shenzhen University School of Medicine, Shenzhen, 518060 China

## Abstract

During China’s urbanization process, rural labor migrants have been suggested to be one important bridge population to change urban-rural distribution on tuberculosis (TB) burden. Aiming to estimate the prevalence of TB infection and to track the active disease development in rural labor migrants, a prospective study was conducted in Shenzhen city, southern China. TB infection was detected using interferon-γ release assay (IGRA). Here we mainly report the characteristics of TB infection in the study population based on the baseline survey. A total of 4,422 eligible participants completed baseline survey in July 2013. QuantiFERON (QFT) positivity rates 17.87% (790/4,422) and was found to be consistent with the local TB epidemic of the areas where the participants immigrated from. Age, smoking, residence registered place, and present of BCG scars were found to be independently associated with QFT positivity. Additionally, evidence for interaction between smoking and age was observed (p for likelihood ratio test < 0.001). Our results suggested that the development of TB control strategy including latent TB infection management should pay more attention to the rural flowing population due to their high mobility and higher prevalence of TB infection.

## Introduction

Tuberculosis (TB) is a top infectious disease killer worldwide. In China, between 1990 and 2010, the prevalence of smear-positive tuberculosis and tuberculosis-related mortality fell by 63% and 80%, respectively^1^. These improvements have primarily been attributed to the nationwide scale-up of a tuberculosis control program using short-courses of directly observed treatment (DOTS). However, despite the progress it has made, China is still facing a serious challenge to achieve the target of the new post-2015 End TB Strategy^2^. This strategy outlines a 2025 milestone of 50% reduction in incidence and 75% reduction in mortality, and an overall 2035 target of 90% reduction in incidence and 95% reduction in mortality. In order to reach these targets, China has been suggested to redouble TB control efforts and adopt new TB control strategies^3^. The management of latent TB infection (LTBI) is a critical component of the new post-2015 End TB Strategy, and WHO issued guidance for upper-middle and high-income countries (including China) with an incidence rate of less than 100 per 100 000 population in 2015^4^. Before a national strategy for systematic testing and treatment of LTBI in the subgroups under high risk of disease development, China needs to clarify the epidemiological profiles of TB infection in different populations^5, 6^.

China’s rapid urbanization process, led by the nationwide economic reforms, created large-scale domestic migrations. In nowadays China, about 260 million Chinese people are living away from where they are formally registered, and the overwhelming majority of them (about 220 million) are rural migrants living and working in urban areas but without formal urban household registration status^7^. Majority of them were not entitled to subsidized housing, education, social security or medical benefits^8, 9^. As a result, urban migrants had a lower social and economic level^10^, a limited knowledge of tuberculosis^11^, and a higher risk of tuberculosis than local residents^12^. Therefore, rural labor migrants have been suggested to be one important bridge population to change urban-rural distribution on TB burden. This prospective study aims to estimate the prevalence of LTBI and to track the active disease development among those infections in rural labor migrants in Shenzhen city, Guangdong province, southern China. Of the 10.4 million residents in Shenzhen, 77% were rural to urban migrants and 46% were factory workers^13^. The research results in Shenzhen city will provide critical reference for the development of suitable LTBI management strategies for urban migrants in the era of urbanization in China.

## Results

The targeted population of the three factories was 4648. Between July and September, 2013, a total of 4,522 individuals were included in the baseline survey. As shown in Supplementary Table 1, 43 were excluded because of self-reported history of PTB and 57 were excluded because of identification of current clinically suspected PTB.

Finally, 4,422 participants were included in the analysis of the prevalence of TB infection. As we compared the gender, age, and residence registered place between the targeted population and actual included population, there was no statistical difference with p values of 0.453, 0.322 and 0.722, respectively (Supplementary Table 2).

The main characteristics of the investigated 4,422 participants are summarized in Table 1. Overall, 60.47% (2,674/4,422) participants were males and almost half of them were aged 20–29 years. 41.25% (1,822/4,422) completed junior high school, and 51.36% (2,271/4,422) were married. The participants were from 28 provinces/autonomous regions (as shown in Supplementary Table 3), and 47.58% (2,104/4,422) of them were resident registered in Middle China. Over a quarter of participants (28.95%, 1,280/4,422) reported ever smoked and 29.40% (1,300/4,422) had ever consumed alcohol. BMI distribution showed that more than a third of the participants were overweight or obese (32.18%, 1,423/4,422). Almost half of participants (45.68%, 2,010/4,422) presented at least one BCG scars. Most of the participants did not report a history of close contact with TB patient (95.82%, 4,237/4,422). 790 (17.87%), 3,611(81.66%), and 21 (0.47%) participants were QFT positive, negative, and indeterminate result, respectively.

**Table 1:** Characteristics of the study population.

Variables	n	%*
Total	4422	100
Gender		
Male	2674	60.47
Female	1748	39.53
Age		29.46 ± 8.53^#^
16–19 years	274	6.20
20–29 years	2431	54.98
30–39 years	1065	24.08
≥40 years	652	14.75
Ethnicity		
Han	4322	97.74
Others	100	2.26
Highest education level		
Primary school or lower	220	4.98
Middle school	1822	41.25
High school	1573	35.61
College and higher	802	18.16
Current marriage status		
Married	2271	51.36
Unmarried	2151	48.64
Household per capita income		
<13000 RMB	517	11.69
≥13000 RMB	3905	88.31
Work type of migrant worker		
Manufacturing	3689	83.42
Administration	466	10.54
Service	267	6.04
Residence registered place		
East China	1094	24.74
Middle China	2104	47.58
West China	1224	27.68
Smoking		
Never smoke	3142	71.05
Current and Ever smoked	1280	28.95
Drinking		
Yes	1300	29.40
No	3122	70.60
BCG scars		
Absent	2402	54.32
Present	2020	45.68
Body mass index		
<18.5	593	13.41
18.5–24	2766	62.65
24–28	830	18.77
≥28	233	5.27
With a history of close contact with TB patient		
No	4237	95.82
Yes	185	4.18
With a history of immune system disease		
No	4394	99.36
Yes	28	0.64
With suspect PTB symptoms		
No	4335	98.03
Yes	87	1.97
QFT-GIT		
Negative	3611	81.66
Positive	790	17.87
Indeterminate	21	0.47

^*^Sum might not always be in total because of missing data. ^#^Means ± standard deviation.

Abbreviation: BCG = Bacillus Calmette-Guerin. BMI = body-mass index. PTB = Pulmonary tuberculosis. TB = Tuberculosis. QFT=QuantiFERON-TB Gold In-Tube.

Table 2 shows the results of QFT by age and gender. There is an increasing trend for QFT positivity with the increasing of age (p for trend < 0.001). Results of association analysis on QFT positivity was showed in Table 3. In the stepwise selectiob multivariate logistic regression analysis, older age (adjusted odds ratio (aOR) = 1.07, 95% confidence interval (CI) = 1.00–1.08), ever smoked (aOR = 1.30, 95% CI = 1.10–1.54), residence registered place in middle (aOR = 1.26, 95% CI = 1.02–1.55) and west of China (aOR = 1.47, 95% CI = 1.17–1.84) were observed to be the factors independently associated with increased QFT positivity. Present of BCG scars was found to be independently association with decreased QFT positivity with an aOR of 0.82 (95% CI = 0.69–0.97).

**Table 2:** Results of QuantiFERON-TB Gold In-Tube (QFT) among study populations by age and gender.

	Total	15–19 years	20–29 years	30–39 years	≥40 years	*p*
Total						
Negative	3611 (81.66)	261 (95.26)	2111 (87.20)	825 (77.46)	414 (63.50)	<0.001
Positive	790 (17.87)	13 (4.74)	310 (12.75)	237 (22.25)	230 (35.28)	
Indeterminate	21 (0.47)	0	10 (0.41)	3 (0.28)	8 (1.23)	
Male						
Negative	2158 (80.70)	132 (94.29)	1287 (86.49)	519 (77.12)	220 (58.98)	<0.001
Positive	509 (19.04)	8 (5.71)	198 (13.31)	154 (22.88)	149 (39.95)	
Indeterminate	7 (0.26)	0	3 (0.20)	0	4 (1.07)	
Female						
Negative	1453 (83.12)	129 (96.27)	824 (87.38)	306 (79.06)	194 (69.53)	<0.001
Positive	281 (16.08)	5 (3.73)	112 (11.88)	83 (21.17)	81 (29.03)	
Indeterminate	14 (0.80)	0	7 (0.74)	3 (0.77)	4 (1.43)

**Table 3:** Potential risk factors associated with QFT positivity.

Variable	n/N (%)	Univariate analysis		Multiple analysis	
		Crude OR (95%CI)	*p*	Adjusted OR (95%CI)	*p*
Gender					
Male	509/2674 (19.04)	Ref.	0.012		
Female	281/1748 (16.08)	0.82 (0.69, 0.96)			
Age	790/4422 (17.87)	1.07 (1.06, 1.08)	<0.001	1.07 (1.06, 1.08)	<0.001
Ethnicity					
Han	776/4322 (17.95)	Ref.	0.310		
Others	14/100 (14.00)	0.93 (0.81, 1.07)			
Education level					
Primary school or lower	61220 (27.73)	Ref.	0.007		
Middle school	324/1822 (17.78)	0.56 (0.41, 0.78)			
High school	278/1573 (17.55)	0.56 (0.40, 0.77)			
College and higher	129/807 (15.99)	0.50 (0.35, 0.70)			
Current marriage status					
Married	539/2271 (23.73)	Ref.	<0.001		
Unmarried	25 1/2151 (11.67)	0.43 (0.36, 0.50)			
Household per capita income					
<13000 RMB	68/517 (12.77)	Ref.	0.001		
≥13000 RMB	724/3905 (18.54)	1.56 (1.19, 2.04)			
Work type of migrant worker					
Manufacturing	637/3689 (17.27)	Ref.	0.051		
Administration	60/267 (22.47)	1.39 (1.03, 1.88)			
Service	93/466 (19.96)	1.20 (0.94, 1.52)			
Smoking					
Never smoked	522/3142 (16.61)	Ref.	0.001	Ref.	
Ever smoked	268/1280 (20.94)	1.33 (1.13, 1.57)		1.30 (1.10,1.54)	0.003
Drinking					
No	540/3122 (17.3)	Ref.	0.126		
Yes	250/1300 (19.23)	1.14 (0.96, 1.34)			
BCG scars					
Absent	403/2402 (16.78)	Ref.	0.040	Ref.	
Present	387/2020 (19.16)	1.18 (1.01, 1.37)		0.82 (0.69, 0.97)	0.019
Body mass index					
<18.5	71/593 (11.97)	1.51 (1.16, 1.98)			
18.5–24	472/2766 (17.06)	Ref.	<0.001		
24–28	198/830 (23.86)	2.30 (1.72, 3.09)			
≥28	49/233 (21.03)	1.96 (1.31, 2.92)			
History of close contact with TB patient					
No	742/4237 (17.51)	Ref.	0.004		
Yes	48/185 (25.95)	1.65 (1.18, 2.31)			
With a history of immune system disease					
No	786/4394 (17.89)	Ref.	0.621		
Yes	4/28 (14.29)	0.77 (0.27, 2.21)			
With suspect PTB symptoms					
No	771/4335 (17.79)	Ref.	0.330		
Yes	19/87 (21.84)	1.29 (0.77, 2.16)			
Residence registered place					
East	163/1094 (14.90)	Ref.	0.003	Ref.	
Middle	385/2104 (18.30)	1.28 (1.05, 1.56)		1.26 (1.02,1.55)	0.030
West	242/1224 (19.77)	1.41 (1.13, 1.75)		1.47 (1.17,1.84)	0.001

Abbreviation: BCG = Bacillus Calmette-Guerin. TB = Tuberculosis. QFT = QuantiFERON-TB Gold In-Tube. OR = odds ratio. CI = Confidence interval.

As shown in Table 4, significant modification effect was observed for age on the relation of smoking with QFT positivity (p for interaction < 0.001). Among the age group 30 years older, smoker was found to be associated with significantly increased risk of TB infection with an aOR of 1.49 (1.19, 1.87). No interaction was observed between BCG vaccination and age or smoking.

**Table 4:** The association of QFT positivity between age, BCG status, and smoking status among study populations.

Variables		QFT positivity (n/N)	%	Adjusted OR (95%CI)	p for likelihood ratio test
***Age and smoking status*** ^***†***^					
16–29 years	Never-smoker	224/1938	11.56	Reference	<0.001
	Ever-smoker	99.767	12.91	1.17 (0.90, 1.50)	
≥30 years	Never-smoker	298/1204	24.75	Reference	
	Ever-smoker	169/513	32.94	1.49 (1.19, 1.87)	
***Age and BCG scar status*** ^***#***^					
16–29 years	Without BCG	230/1719	13.38	Reference	0.583
	With BCG	93/986	9.43	0.67 (0.52, 0.86)	
≥30 years	Without BCG	173/683	25.33	Reference	
	With BCG	294/1034	28.43	1.16 (0.93, 1.45)	
***Smoking and BCG scar status*** ^***&***^					
Never-smoker	Absent	281/1756	16.00	Reference	0.067
	Present	241/1386	17.39	0.87 (0.71, 1.06)	
Ever-smoker	Absent	122/646	18.89	Reference	
	Present	146/634	23.03	1.02 (0.77, 1.35)	

Abbreviations: BCG = Bacillus Calmette-Guerin; CI = confidence interval; OR = odds ratio; QFT = QuantiFERON-TB Gold In-Tube.

^†^Adjusted for BCG status.

^#^Adjusted for smoking status.

^***&***^Adjusted for age.

The cumulative impact of multiple selected risk factors that associated with QFT positivity in the univariate analyses was estimated in Supplementary Table 4 (gender of male, age ≥20 years, middle school or higher education level, present or ever married, household per capita income over ≥13000 RMB, present or ever smoking, with BCG scars, BMI <18.5, with a history of close contact with TB patient, and residence registered place not in east China). An increasing risk of QFT positivity was observed with the increasing number of presented factors in the study participants (p for trend < 0.001).

## Discussion

This present study aims to estimate the prevalence and associated factors of TB infection among rural labor migrants in Shenzhen city, southern China. Among 4,422 participants enrolled from the light industry, 790 were QFT positive with a prevalence of 17.87%. QFT positivity was found to be consistent with the local TB epidemic of the place where the participants registered residence. Age, smoking, residence registered place, and present of BCG scars were found to be independently associated with QFT positivity.

In our previously published study addressing in TB infection in rural China^14, 15^, the age and gender standardized prevalence of QFT positivity was found to be ranged from 13.5% to 19.8% in the selected study sites with various TB epidemic. However, it only included the registered residents with continuous residence at the study site for ≥6 months over the past year before survey. Therefore, many individuals in the 20–40 age range were not recruited because they have generally moved to work in urban areas. Hence it is reasonable to speculate that these rural labor migrants might change TB epidemic in the cities, as well, their health problems including TB infection and active disease development might be changed in the circumstance of the living city. For the first time, as we know, the present study provided direct evidences to support that the prevalence of TB infection in rural labor migrants was determined by the epidemic status of the place where they migrated from. In 2000, the Fourth National Tuberculosis Epidemiology Survey in China showed that the prevalence of tuberculosis infection (defined by a tuberculin skin-test induration ≥6 mm) was 44.5% for all age groups^16^. With the availability of IGRA, the prevalence of LTBI has recently been investigated using the new method in specific populations in China such as students^17^, TB close contacts^18^, and healthcare workers^19^. Based on the evidence that the performance of TST might be affected by various factors including BCG vaccination and exposure to non-tuberculous mycobacteria, the prevalence of LTBI in China might be overestimated by tuberculin skin-test as compared to IGRA^14, 20^. As a result, testing of high risk populations and prophylactic treatment of those with LTBI becomes to be feasible in China.

QFT positivity among the participants migrated from West, Middle, and East China was observed to be 14.9%. 18.3%, and 19.8%, respectively. It is consistent with the fact that the burden of TB was increasing from East China to West China^21, 22^. Considering TB prevalence in rural areas in China was approximately twice as high as it in urban areas and Shenzhen city locates in the area with low prevalence of TB^15^, it is not surprising that the higher prevalence of TB infection and of active disease among rural labor migrants might influence TB epidemic in local residents^23^. As compared to those residents continuously living in rural areas, we do not know whether the changed living conditions of rural labor migrants would change their risk of disease developing from infection^24, 25^. Long term follow-up studies with larger sample size are needed to disclose such an important scientific question. However, the high population mobility, as observed in the present study, makes it more difficult. Even so, TB control strategy development including LTBI management should pay attention to the rural flowing population in the era of urbanization in China^26, 27^.

A significant association between smoking and QFT positivity was observed in our study population. Such an association has been reported previously in various populations including rural population in China^28, 29^. The adverse effects of smoking on pulmonary immunity might explain the susceptibility of smoking population to TB infection^30, 31^. Furthermore, smoking is found to be associated not only with TB infection and disease development, but also with delayed bacteriologic clearance, recurrence and TB related deaths^32–34^. Therefore, it has been proposed that tuberculosis control policies should incorporate tobacco control as a preventive intervention to conquer there two co-prevalent major alarming global health issues^35^. Meanwhile, the risk of QFT positivity in different participant’s residence registered place in our study shows escalating trend from 1.26 to 1.47 (Table 3). We noted this trend across all three regions is similar trends for the local economy level. This finding could be due to some patterns of life may different in different economy level regions, such as education level and household per capita income.

Consistently, the evidence of effect modification between age and smoking were found in this study^36^. Higher risk of TB infection observed for elderly might be partly explained by their longer duration and/or higher intensity in cigarette smoking and the related lung structure impairment. In addition, our results indicated that there might be a cumulative risk of TB infection when a person was exposed to more risk factors. Therefore, in China rural community, such risk combinations might be helpful to identify subgroups under higher risk of TB infection for further LTBI screening and management.

When interpreting the results, limitations of this study should be kept in mind. First, we selected light industry labors as study population, however they could not represent all rural labor migrates living in Shenzhen City. It is logical to imagine that the types of work might be related with the risk of TB disease and exposure to infection. Second, potential bias caused by infection status misclassification based on IGRA could not being excluded due to missing golden standard method to test TB infection. Third, association between the risk of infection and exposures with lower prevalence in the study population, such as close contacts, could not be identified with a reliable study power because of the limited number of QFT positives observed in our study.

In summary, our results suggested the prevalence of TB infection in the rural labor migrates was determined by the epidemic status of the place where they migrated from. It is reasonable to speculate that rural labor migrants could be one important bridge population to change current TB transmission patterns in China. TB control strategy development including LTBI management should pay attention to the rural flowing population in the stage of speed-up urbanization in China.

## Methods

### Study design

The present study was based on the baseline survey of a prospective cohort study^14^ conducted in rural labor migrants in Shenzhen city, China. The study was organized by the Institute of Pathogen Biology of Chinese Academy of Medical Sciences (IPB, CAMS) and Shenzhen Third People’s Hospital. The study encompasses a total of three years (2013–2015). The aim of the baseline survey (2013) was to detect TB infection for each participant using an interferon-γ release assay (IGRA) after excluding those with current TB or with a history of TB. During the follow-up phase (-2015), those identified infections at baseline would be followed to observe the development of active TB through active case finding. Westat (Rockville, Maryland, USA) provided independent monitoring during the study implements.

### Study Population

The baseline survey followed a clustering sampling design. Three factories were purposively selected in the considerations of representativeness to type of factories (mainly high tech and electronic products), willingness to participation and capacity in coordinating field investigation and testing. The factories produced high technology products (4,964 workers), television sets (1,560 workers) and electronic products (1,761workers). The study population hence only included factory workers of the light industry.

Rural to urban labors working at the selected factories were selected as the target population of the present study. The inclusion criteria were: birth prior to 1 June, 1998 (≥15 years old); coming from other areas of China and was not an official registered resident in Shenzhen; continuous residence in Shenzhen for ≥6 months over the past year; able to complete the investigations and tests during the study duration; provision of voluntary written informed consent. The exclusion criteria were: current active TB; self-reported history of TB; pregnancy.

### Ethical Review

The study protocol was approved by the ethics committees of IPB, CAMS. Upon explanation of the study protocol, written informed consent was obtained from the participant for all study participants.

All experiments and methods in our study were approved by the ethics committees of the IPB, CAMS. Meanwhile, all experiments and methods were carried out according to the approved guidelines above.

### Study procedures

The baseline survey was conducted during July to September 2013. For each study participant, socio-demographic information was collected by a standardized questionnaire administered by trained interviewers. Data collected included ethnicity, educational level, occupation, marital status, household per capita income in 2012 (i.e. total family income/number of people in the household), area of household living space, smoking and alcohol consumption status. In addition, current TB status and history of reported TB disease, history of close contacts, history of immune suppression and chronic diseases were assessed. Information on history of current and previous TB was verified with the national active TB case report system. Suspected pulmonary TB (PTB) symptoms were obtained and evaluated by physicians. Height, weight, pulse and the presence of a Bacillus Calmette-Guerin (BCG) scar were examined as well. Blood biochemical examinations were provided for free to encourage participation.

Venous blood was collected for QuantiFERON-TB Gold In-Tube (QFT, QIAGEN, USA) testing, one commercially available IGRA. QFT was performed as recommended by the manufacturer using a cutoff value of ≥0.35 IU/mL. Digital chest radiography (CXR) was performed for all study participants.

Participants with symptoms suggestive of PTB or with radiographic abnormalities consistent with active PTB were transferred for disease confirmation according to WHO guidelines. Individuals with sputum smear or culture positive PTB, or with suspected PTB (defined by radiographic abnormalities consistent with active PTB together with LTBI) were not included in the LTBI analysis.

### Data management and statistical analysis

Questionnaire data, physical examination data (height, weight, pulse, and presence of BCG scar) and laboratory results (QFT and blood biochemical examination) were double entered into a spreadsheet and checked by web-based project-specific data collection and management software. After cleaning, the data were then converted and analyzed using Statistical Analysis System (SAS 9.2; SAS Institute Inc., NC, USA).

To identify potential variables related with QFT, univariate analysis was performed using Pearson’s chi-square test. Stepwise backward multiple logistic regression analysis was then used to identify factors that were independently associated with QFT positivity. The significance level for factors to stay in the model was 0.05. Odds ratios (OR) were calculated, as appropriate along with 95% confidence intervals (CI). Evidence for interaction between factors was assessed using the likelihood ratio test (LRT) by comparing logistic regression models with and without an interaction term.

## Electronic supplementary material

Supplementary files Online Content Only
