# Supplementary material for: Tuberculosis infection in rural labor migrants in Shenzhen, China: Emerging challenge to tuberculosis control during urbanization
Source: Sci Rep. 2017 Jun 30;7:4457. doi: 10.1038/s41598-017-04788-1 (PMC5493641; doi:10.1038/s41598-017-04788-1)
Supplement: Supplementary file 1 — Supplementary files Online Content Only [file 41598_2017_4788_MOESM1_ESM.doc]

**Tuberculosis infection in rural labor migrants in Shenzhen, China: Emerging challenge to tuberculosis control during urbanization**

Xiangwei Li,^1^* Qianting Yang,^2^* Boxuan Feng,^1^ Henan Xin,^1^ MingXia Zhang,^2^ Qunyi Deng,^2^ Guofang Deng,^2^ Wanshui Shan,^2^ Jianrong Yue,^2^ Haoran Zhang,^1^ Mufei Li,^1^ Hengjing Li,^1^ Qi Jin,^1^ Xinchun Chen,^2,3^* Lei Gao^1^*

1. MOH Key Laboratory of Systems Biology of Pathogens, Institute of Pathogen Biology, and Center for Tuberculosis, Chinese Academy of Medical Sciences and Peking Union Medical College, Beijing, 100730, China

2. Guangdong Key Laboratory for Emerging Infectious Diseases, Shenzhen Key Laboratory of Infection & Immunity, Shenzhen Third People's Hospital, China

3. Department of Pathogen Biology, Shenzhen University School of Medicine, Shenzhen, 518060, China

*These authors contributed equally

**Running title:** TB infection in rural labor migrants

**Correspondence:**

Prof. Lei Gao, Institute of Pathogen Biology, CAMS & PUMC. Dong Dan San Tiao 9. Beijing, 100730, China. Email: gaolei@ipbcams.ac.cn. Prof. Xinchun Chen, Department of Pathogen Biology, Shenzhen University School of Medicine, Shenzhen, 518060, China. Email: chenxinchun@163.com.

**Word count:** 188 in the abstract; 2651 in the text

**Supplementary table 1. Exclusion of e**ligible population included in the survey

| Eligible population included in the baseline survey | **4522** |
| --- | --- |
| Excluded because of self-reported history of tuberculosis | **43** |
| Excluded because of present clinically suspected pulmonary tuberculosis† | **57** |
| Actual population assessed for the prevalence of latent tuberculosis infection | **4422** |

†If digital chest radiography abnormal and results for interferon-γ release assays were positive or tuberculin skin tests were strong positive (i.e., induration diameter ≥15 mm or presence of blister or necrosis), the case was identified as with clinically suspected pulmonary tuberculosis.

**Supplementary table 2. Population sampling among the study sites**

| **Variables** | **Total** | **Actual population included** | **%** | ***p*** |
| --- | --- | --- | --- | --- |
| Total | 4648 | 4422 | 95.14 | */* |
| Gender |  |  |  | 0.453 |
| Male | 2805 | 2674 | 95.33 |  |
| Female | 1843 | 1748 | 94.85 |  |
| Age |  |  |  | 0.322 |
| 16-19 years | 288 | 274 | 95.14 |  |
| 20-29 years | 2542 | 2431 | 95.63 |  |
| 30-39 years | 1130 | 1065 | 94.25 |  |
| ≥ 40 years | 688 | 652 | 94.77 |  |
| Ethnicity |  |  |  | 0.067 |
| Han | 4547 | 4322 | 95.05 |  |
| Others | 101 | 100 | 99.01 |  |
| Work type of migrant worker |  |  |  | 0.001 |
| Manufacturing | 3776 | 3689 | 97.70 |  |
| Administration | 271 | 267 | 98.52 |  |
| Service | 601 | 466 | 77.54 |  |
| [Residence registered](http://dict.cn/place where his residence is registered) place |  |  |  | 0.732 |
| East China | 1155 | 1094 | 94.72 |  |
| Middle China | 2207 | 2104 | 95.33 |  |
| West China | 1286 | 1224 | 95.18 |  |

**Supplementary Table 3. The study participants were found to be migrating from 28 provinces**.

| **Province** | **n** | **%** | **District*** |
| --- | --- | --- | --- |
| Anhui | 71 | 1.61% | East China |
| Heilongjiang | 17 | 0.38% | Northeast China |
| Jilin | 12 | 0.27% | Northeast China |
| Liaoning | 12 | 0.27% | Northeast China |
| Inner Mongolia | 13 | 0.29% | North China |
| Xinjiang | 1 | 0.02% | Northeast China |
| Gansu | 45 | 1.02% | Northeast China |
| Qinghai | 1 | 0.02% | Northeast China |
| Ningxia | 3 | 0.07% | Northeast China |
| Shaanxi | 144 | 3.26% | Northwest China |
| Shanxi | 7 | 0.16% | North China |
| Hebei | 13 | 0.29% | North China |
| Shandong | 33 | 0.75% | East China |
| Henan | 255 | 5.77% | Central China |
| Jiangsu | 14 | 0.32% | East China |
| Zhejiang | 3 | 0.07% | East China |
| Hubei | 617 | 13.95% | Central China |
| Hunan | 737 | 16.67% | Central China |
| Sichuan | 311 | 7.03% | Southwest China |
| Chongqing | 59 | 1.33% | Southwest China |
| Yunnan | 87 | 1.97% | Southwest China |
| Guangxi | 518 | 11.71% | South China |
| Jiangxi | 405 | 9.16% | East China |
| Fujian | 14 | 0.32% | East China |
| Hainan | 24 | 0.54% | South China |
| Tianjin | 1 | 0.02% | North China |
| Guangdong | 902 | 20.40% | South China |
| Guizhou | 104 | 2.35% | Southwest China |

*The provinces were divided into seven districts by socioeconomics level from national bureau of statistics of the People’s Republic of China

**Supplementary Table 4. Combination effect of potential factors on the risk of tuberculosis infection**

| Number of factors | **n/N (%)** | **OR (95%CI)** | **p for trend** |
| --- | --- | --- | --- |
| ≤3 | 5/95 (5.26) | Ref. | <0.001 |
| 4 | 28/299(9.36) | 1.86 (0.70, 4.96) |  |
| 5 | 121/914 (13.24) | 2.75 (1.09, 6.90) |  |
| 6 | 224/1314 (17.05) | 3.70 (1.49, 9.21) |  |
| 7 | 238/1093 (21.77) | 5.01 (2.01, 12.47) |  |
| 8 | 143/578 (24.74) | 5.92 (2.36, 14.85) |  |
| 9 or 10 | 31/129 (24.03) | 5.69 (2.12, 15.28) |  |

Factors：Sex (female=0, male=1), Age (＜20=0, ≥20=1), Education level (Primary school or lower=1, other=0), Current marriage status (Unmarried=0, married=1), Household per capita income (<13000 RMB=0, ≥13000 RMB=1), Smoking (never smoking=0,ever smoking=1), Number of BCG scars (no=1, yes=0), BMI (≥18.5=0, ＜18.5=1), History of close contact with TB patient (no=0, yes=1), Residence registered place (east=0, other=1).

CI: confidence interval; OR=odds ratio; N/A, not available.


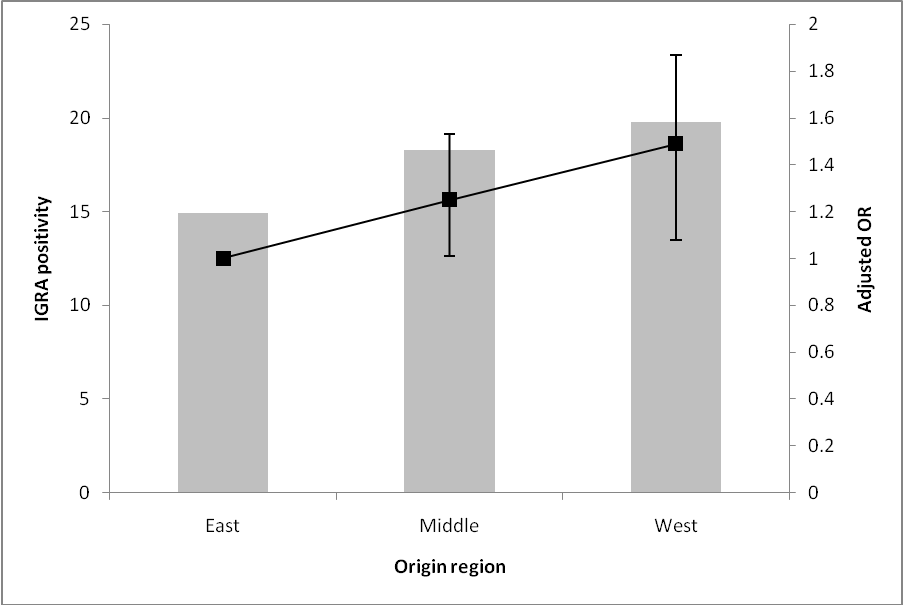


Supplementary figure 1. The prevelance of QFT pisitivity in the study participants subgrouped by the place of their residence registration.
